# The Paraspinal Sarcopenia at the Upper Instrumented Vertebra Is a Predictor of Discoligamentous but Not Bony Proximal Junctional Kyphosis

**DOI:** 10.3390/jcm14041207

**Published:** 2025-02-12

**Authors:** Zach Pennington, Anthony L. Mikula, Abdelrahman Hamouda, Maria Astudillo Potes, Ahmad Nassr, Brett A. Freedman, Arjun S. Sebastian, Jeremy L. Fogelson, Benjamin D. Elder

**Affiliations:** 1 Department of Neurologic Surgery, Mayo Clinic, Rochester, MN 55905, USA; 2 Department of Neurosurgery, University of California San Francisco, San Francisco, CA 94143, USA; 3 Department of Orthopaedic Surgery, Mayo Clinic, Rochester, MN 55905, USA

**Keywords:** Hounsfield units, bone quality, vertebral bone quality (VBQ), adult spinal deformity, sarcopenia, proximal junctional kyphosis

## Abstract

**Background/Objectives**: Both poor bone quality and paraspinal sarcopenia have been suggested as risk factors for proximal junctional kyphosis (PJK) at the upper instrumented vertebra (UIV) following long-segment thoracolumbar fusion. **Methods**: Adults ≥50 with a T1-6 UIV were identified, and data were gathered on pre- and postoperative spinopelvic parameters, bone quality (using Hounsfield units and vertebral bone quality score), and paraspinal cross-sectional area at L3 and the UIV. PJK was defined by a ≥10° increase in the proximal junctional angle. Cox regressions were performed to identify PJK risk factors; PJK was subdivided into types 1–3 based on the Yagi–Boachie classification. **Results**: In total, 15/76 patients (median age 66; 72.4% female) experienced PJK; 10 experienced type 1, 4 experienced type 2, and one experienced type 3. Univariable Cox regression showed that PJK was negatively correlated with total paraspinal muscle CSA at the UIV (HR 0.74/100 mm^2^; 95% CI [0.57, 0.6]; *p* = 0.02). Lower total paraspinal CSA at L3 (HR 0.94/100 mm^2^; *p* = 0.07) and higher postoperative global tilt (HR 1.03; *p* = 0.09) also trended toward significance. Similarly, type 1 PJK was predicted by smaller total paraspinal CSA at the UIV (HR 0.64/100 mm^2^; [0.45, 0.92]; *p* = 0.02). Paraspinal CSA was not predictive of type 2 PJK, but lower HU at the UIV and UIV + 1 trended toward significance (HR 0.98/unit; *p* = 0.16). A comparison of type 1 and 2 PJK showed a higher average of paraspinal CSA and a lower average of HU at the UIV. **Conclusions**: Global alignment and paraspinal sarcopenia were most predictive of PJK, though paraspinal sarcopenia was only predictive of type 1. Type 2 may be better predicted by bone quality.

## 1. Introduction

Long-segment thoracolumbar instrumented fusion is considered a definitive treatment for symptomatic adult spinal deformity (ASD), causing persistent disability despite nonoperative management. While most ASD patients do not require operative intervention, the high prevalence of elderly patients with ASD (nearly two-thirds of patients over the age of 60) [1] means that the absolute number of patients with operative indications is large. One of the most common delayed complications of such long-segment fusions is proximal junctional kyphosis (PJK), which has been reported to affect anywhere between 5 and 61% of patients [2]. Of these, 10–31% of cases will require operative intervention [3] with a per-event cost of approximately $56,000 [4].

The exact pathogenesis of PJK is not completely understood, and risk factors for the occurrence of PJK have been the subject of many clinical studies [5]. It is thought that PJK results from the funneling of stresses to the motion segment suprajacent to the fusion construct secondary to the immobilization of all motion segments below it [6,7]. To this end, Dubousset and Diebo [8] recently reviewed the extant knowledge on PJK, framing it in the context of biological and mechanical causes, the latter of which can be grouped into local and global phenomena. Local mechanical causes include soft tissue disruption, weakness of the posterior instrumentation, and structural incompetence/weakness of the anterior column. Weakness of the instrumentation can lead to failure to maintain correction, weakness of the anterior column can result in compression fractures with resultant deformity, and disruption of soft tissue weakness in the posterior tension band, which can lead to gradual kyphosis. Global mechanical causes include anything that alters the overall balance, so as to displace the patient’s head from the gravity line running through the “pelvic vertebra” and disrupting horizontal gaze. This may include selection of an unstable (e.g., junctional) vertebra as the upper instrumented vertebra (UIV), overly aggressive correction of lumbar lordosis, or inducing hypokyphosis in the thoracic spine, which will induce a reflexive cervicothoracic kyphosis. Biological causes include age-related muscle atrophy (sarcopenia), poor underlying bone health, and neuromuscular pathologies (e.g., Parkinson’s disease, which causes camptocormia).

Along these lines, Yagi and Boachie [9] described PJK as being potentially attributable to three etiologies—discoligamentous failure, bony failure, and failure of bone/implant interface. Others have similarly investigated the potential influence of bone integrity [10,11], soft tissue integrity, and paraspinal muscle bulk as potential predictors of PJK [6,12]. One finite element analysis by Cammarata and colleagues [7] found that injury to the bilateral facet capsules at the UIV/UIV + 1 junction (from bilateral complete inferior facetectomy) and dissection of the supraspinous and interspinous ligaments at the UIV/UIV + 1 lead to a 53% increase in the proximal junctional angle and 83% increase in flexional moment. They also found the use of transverse process hooks [versus pedicle screws] at the UIV to decrease the junctional stresses. However, they did not consider the relative contribution of the paraspinal musculature, which Dubousset and Diebo [8] highlighted as a potential local mechanical cause of PJK, and which likely contributes to type 1 PJK in the Yagi and Boachie system. To date, most studies that have considered paraspinal muscle bulk as a risk factor for PJK have considered only multifidus cross-sectional area (CSA) when evaluating paraspinal sarcopenia [13] and have similarly evaluated multifidus CSA within the lumbar spine rather than at the UIV. As muscular attachments to the posterior elements of the rostral vertebra exert forces to counteract the kyphosing forces of gravity at the UIV, consideration of total paraspinal CSA at the UIV would seem to be the best surrogate for soft tissue integrity. The relative interplay between paraspinal sarcopenia and underlying bone quality may also influence the failure mode in patients undergoing long-segment thoracolumbar fusion. Accordingly, the objective of the present study was to employ a time-to-event analysis to identify risk factors for PJK following thoracolumbar instrumented fusion for ASD. Additionally, we sought to determine whether the relative predictive value of underlying bone quality and UIV sarcopenia varied depending upon the mode of PJK seen.

## 2. Materials and Methods

After obtaining IRB approval (18-002622) we retrospectively identified patients who underwent long-segment thoracolumbosacral fusion for ASD over an 11-year period at a single tertiary care center with a UIV in the upper thoracic spine (defined as T1-6). Patients were included if (1) they were ≥50 years old at the time of surgery, (2) had preoperative radiographs with features consistent with ASD as defined by the Schwab criteria: coronal Cobb angle ≥ 20°, sagittal vertical axis (SVA) ≥ 5 cm, thoracic kyphosis ≥ 60°, or pelvic tilt ≥ 25°, and (3) they underwent instrumented fusion with distal fixation to the pelvis and UIV in the upper thoracic spine (T1-6). Patients were excluded if (1) they underwent surgery for indications of trauma, infection, or tumor, (2) did not have preoperative CT and MR, and (3) did not have pre- and postoperative upright scoliosis radiographs with a minimum 12-month follow-up.

**Figure 1:**
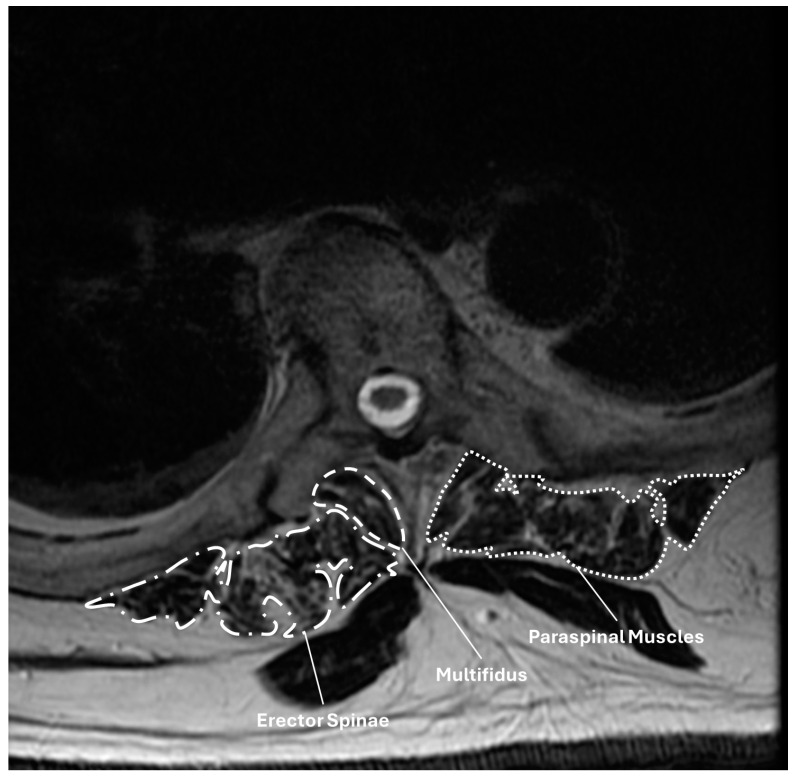
Axial T2-weight MR sequence illustrating the measurement of muscle cross-sectional areas at the upper instrumented vertebra (UIV) for a patient who underwent T5-pelvis fusion. Regions of interest are shown around the multifidus, erector spinae, and aggregate paraspinal musculature.

The charts of patients meeting inclusion/exclusion criteria were then queried for data on demographics (age, sex, insurance type, smoking status), medical comorbidities (Charlson Comorbidity Index/CCI, 5-item modified Frailty Index/mFI5, and American Society of Anesthesiologist (ASA) classification), nutritional status (preoperative albumin), pre- and postoperative spinopelvic parameters, bone quality, and paraspinal musculature cross-sectional area. Spinopelvic parameters included pelvic incidence (PI), pelvic tilt (PT), L1-S1 lumbar lordosis (L1-S1 LL), L4-S1 lumbar lordosis (L4-S1 LL), PI-LL mismatch (PI-LL), sacral slope (SS), pelvic tilt (PT), sagittal vertical axis (SVA), T1-pelvic angle (T1PA), and global tilt. Radiographic parameters were measured by two trained resident surgeons (ZP, ALM) trained by the senior author (BDE). Measures were performed independently by the two reviewers, and the average value was used. Discrepancies >3° were resolved by the senior author (BDE). Bone quality was assessed using Hounsfield units and the Vertebral Bone Quality (VBQ) score. Hounsfield units were measured for the UIV and suprajacent vertebra (UIV + 1) using axial ROIs placed in the mid-body, immediately caudal to the superior endplate, and immediately cranial to the inferior endplate. The HU used in the analysis was the average of these three values as previously described [10,11,14,15]. The VBQ was a modified version of the VBQ score originally described by Ehresman et al. [16]. This modified VBQ used ROIs placed in the medullary bone of the four contiguous vertebrae spanning from UIV + 2 to UIV − 1 on mid-sagittal, non-contrast-enhanced T1-weight sequences. The score was calculated by dividing the median pixel intensity of these four vertebral bodies by the pixel intensity of the cerebrospinal fluid at the same level. Paraspinal musculature was measured on non-contrast-enhanced T2-weighted sequences as described by Pinter et al. [12] (Figure 1). This was performed on axial slices at the mid-L3 level and through the mid-body of the UIV. ROIs were placed on the multifidus and erector spinae (a combination of longissimus and iliocostalis). Total paraspinal musculature cross-sectional area (CSA) was defined as the sum of the multifidus and erector spinae CSA.

### Statistical Analysis

**Figure 2:**
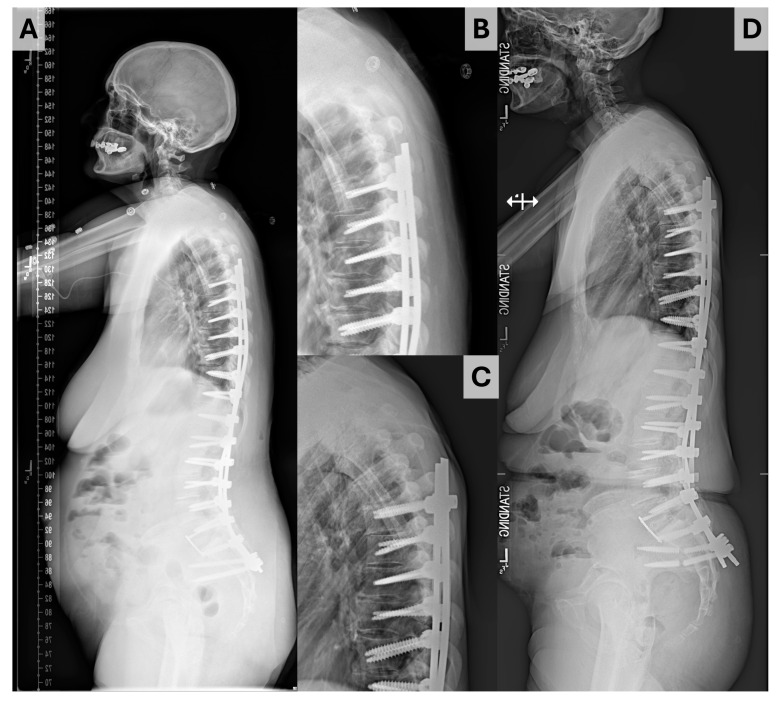
Illustration of Yagi–Boache type 1 PJK. (**A**) Upright immediate postoperative radiograph. (**B**,**C**) Enlarged radiographs illustrating the proximal junction of the construct on immediate postoperative radiograph (**B**) and at the time of PJK diagnosis (**C**). (**D**) Upright postoperative radiograph obtained at the time of PJK diagnosis.

**Figure 3:**
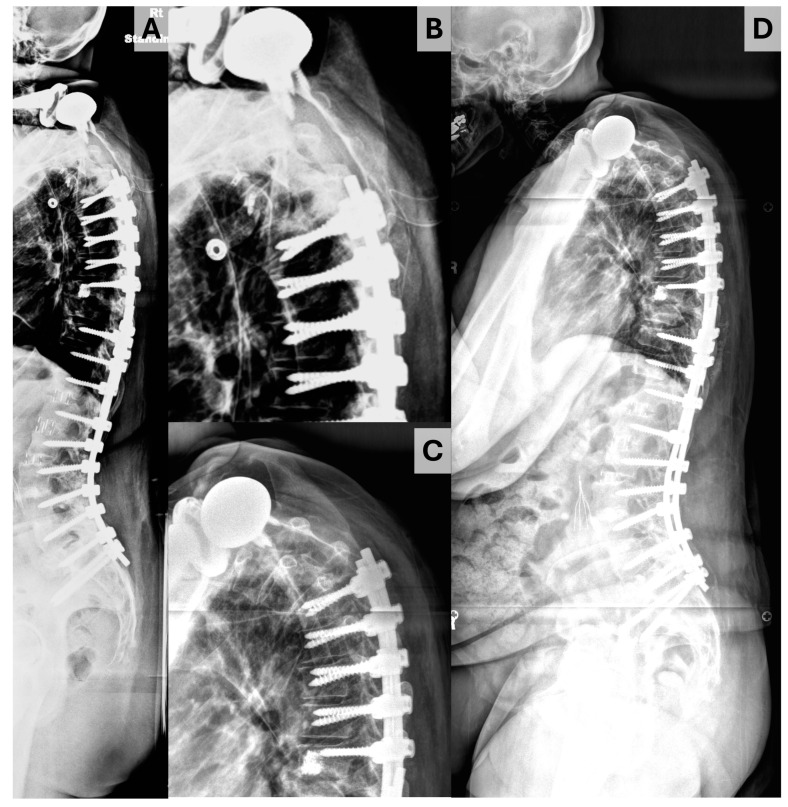
Illustration of Yagi–Boache type 2 PJK. (**A**) Upright immediate postoperative radiograph. (**B**,**C**) Enlarged radiographs illustrating the proximal junction of the construct on immediate postoperative radiograph (**B**) and at the time of PJK diagnosis (**C**). (**D**) Upright postoperative radiograph obtained at the time of PJK diagnosis.

**Figure 4:**
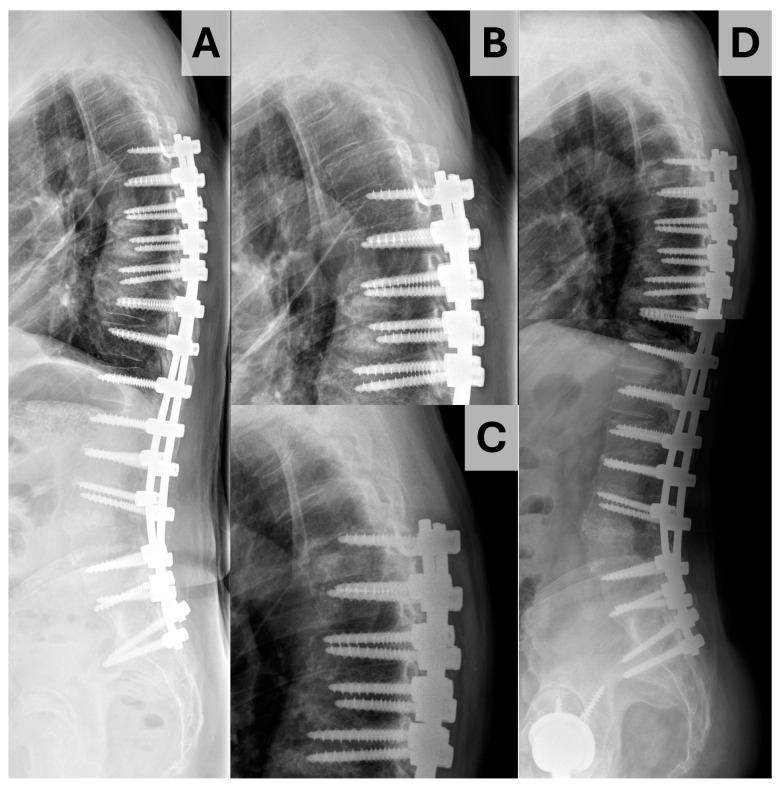
Illustration of Yagi–Boache type 3 PJK. (**A**) Upright immediate postoperative radiograph. (**B**,**C**) Enlarged radiographs illustrating the proximal junction of the construct on immediate postoperative radiograph (**B**) and at the time of PJK diagnosis (**C**). (**D**) Upright postoperative radiograph obtained at the time of PJK diagnosis.

Data were gathered using Microsoft Excel (Redmond, WA, USA) and analyzed using SPSS version 28.0.0 (IBM corporation, Armonk, NY, USA). Descriptive statistics are presented with median and interquartile range (IQR) for continuous data and counts with proportions for discrete data. The primary outcome of interest was proximal junctional kyphosis, which was defined by an increase of ≥10° in the proximal junctional angle (PJA) inscribed by the inferior endplate of the UIV and superior endplate of the suprajacent vertebra (the UIV + 1) [11]. PJK was subdivided into three types using the Yagi–Boachie classification [9]. Type 1 was defined by disk and ligamentous failure (Figure 2), type 2 by bony failure (e.g., wedge compression fracture of the UIV or UIV + 1, Figure 3), and type 3 by implant/bone interface failure (e.g., screw pullout/toggle out, Figure 4). Univariable comparison of patients who did and did not experience PJK was performed using the Mann–Whitney U test for continuous variables and Fisher exact tests for dichotomous variables. To account for the impact of follow-up on the risk of PJK, Cox proportional hazard analysis was performed to identify significant predictors of PJK based on postoperative radiographs, baseline bone quality, and paraspinal muscularity. Those variables identified as significant at the *p* < 0.10 level were introduced to multivariable Cox proportional hazards analysis, with variables showing significance at the *p* < 0.05 level being maintained in the final model. Independent sub-analyses were performed for patients experiencing type 1 and type 2 PJK; analysis was not performed for type 3 PJK as only one patient ultimately experienced this event.

## 3. Results

We identified 152 patients who underwent thoracolumbar instrumented fusion during the queried period, of whom 80 were instrumented to the upper thoracic spine (T1-6). Of these, four patients had insufficient follow-up, leaving 76 patients meeting inclusion/exclusion criteria with a median age of 66.0 yr (IQR 61.0, 71.0 yr) and 72.4% being female (Table 1). At baseline, the mean spinopelvic parameters were as follows: SVA 9.3 cm (4.1, 13.9), PI 54.1° (43.4, 62.9°), PT 30.1° (23.0, 37.4°), L1-S1 LL 31.8° (14.3, 45.6), and T1PA 31.6° (23.3, 40.8). Overall, 15 patients experienced PJK (19.7%), with ten experiencing type 1 PJK, four experiencing type 2, and one experiencing type 3. Univariable comparison (Table 1) of patients who did and did not experience PJK showed patients experiencing PJK to have significantly higher PT on immediate postoperative upright radiographs (25.8° (19.2, 29.1) vs. 19.1° (13.4, 24.8); *p* = 0.04), higher T1PA on immediate postoperative upright radiographs (21.6° (16.1, 28.9) vs. 15.0° (9.5, 21.0); *p* = 0.04) and smaller total paraspinal muscle CSA at the UIV (833 mm^2^ [704, 1050] vs. 964 mm^2^ [859, 1227]; *p* = 0.04). CSA of the multifidus did not differ at L3 or the UIV, and total paraspinal CSA at the mid-L3 level approached but did not meet the threshold of significance (*p* = 0.07).

On univariable Cox proportional hazard analysis (Table 2), time to PJK was found to be positively predicted only by total paraspinal CSA at the UIV (HR 0.74 per 100 mm^2^; 95% CI [0.57, 0.96]; *p* = 0.02) though global tilt (HR 1.03 per °; [1.00, 1.05]; *p* = 0.09) and total paraspinal muscle CSA at the mid-L3 level (HR 0.94 per 100 mm^2^; [0.89, 1.01]; *p* = 0.07) both approached significance. On the multivariable Cox proportional hazards model (Table 3), only total paraspinal CSA at the UIV remained significant (HR 0.74 per 100 mm^2^; [0.57, 0.96]; *p* = 0.03). Analogous univariable Cox regression for type 1 and type 2 PJK (Table 2) showed that total paraspinal CSA at the UIV was predictive only of type 1 PJK (HR 0.64 per 100 mm^2^; [0.45, 0.92]; *p* = 0.02). It was not predictive of type 2 PJK (*p* = 0.32). Increased T1PA (HR 1.12 per °; *p* = 0.07) and poorer bone quality, as measured by the HU of the UIV and UIV + 1 (HR 0.98 per unit; *p* = 0.16), both approached but did not reach the threshold of significance. A direct comparison of patients experiencing type 1 and 2 PJK (Table 4) showed a lower median time to PJK for those experiencing type 1 PJK (932 vs. 93d); however, the difference did not reach statistical significance. Similarly, those experiencing type 1 PJK had non-significantly smaller total CSA for the paraspinal musculature and multifidus alone at the UIV, whereas the average HU of the UIV, UIV + 1, and combination of UIV and UIV + 1 was lower in those experiencing type 2 PJK.

## 4. Discussion

Proximal junctional kyphosis (PJK) is a mechanical complication commonly seen following long-segment thoracolumbar instrumented fusion for adult spinal deformity. Given the high potential cost associated with surgical revision for PJK—estimated by Theologis et al. [4] as $56,000 per event—there is great interest in identifying risk factors for PJK. Various parameters have been advanced as potential risk factors, including persistent postoperative sagittal malalignment, poor bone quality, and atrophy of the paraspinal musculature (paraspinal muscle sarcopenia) [13]. In the present analysis, we evaluated the relative importance of these three classes of parameters in determining both the overall risk of PJK and the type of PJK seen. The results found that paraspinal muscle sarcopenia at the UIV was the strongest predictor of PJK and that the majority of PJK events observed were Yagi–Boachie type 1 PJK (discoligamentous PJK) [9]. Interestingly, when PJK was divided by type, UIV paraspinal muscle atrophy was predictive only of type 1 PJK, consistent with the argument that this subtype of PJK stems from the failure of the supporting posterolateral soft tissue complex. Poor postoperative alignment trended toward being predictive of any type of PJK and in sub-analyses of both type 1 and type 2 PJK; however, the results were not significant, likely due to sample size. Bone quality similarly trended toward predicting the occurrence of type 2 PJK, but not type 1 PJK, consistent with the hypothesis that type 2 PJK results from the insufficient load-bearing capacity of the UIV or UIV + 1. However, the result did not reach significance, again likely due to the relatively small sample size. We also examined the relative impact of overall body habitus (measured by BMI) and nutritional status (measured by albumin level) on the risk of PJK development. We found that neither of these attributes predicted PJK risk, which suggests that PJK is not a complication of poor nutritional status or a complication seen exclusively among overweight patients. We additionally note that while absolute paraspinal muscle CSA predicted type 2 PJK occurrence, it was independent of BMI, suggesting that the morphometric association is likely specific to the paraspinal muscles, as opposed to an overall difference in the patient frame. Additionally, socioeconomic factors, as encapsulated by patient insurance status, were not predictive of PJK, highlighting this complication as a biomechanical consequence of instrumented fusion.

### 4.1. Sarcopenia and PJK

Type 1 PJK, as defined by Yagi et al. [9], describes PJK resulting from the failure of the discoligamentous structures at the motion segment immediately cephalad to the UIV. Specifically, the torques exerted on the posterior elements by the extensor musculature (multifidus and erector spinae) are insufficient to resist the kyphosing torques generated by gravity. As the forces exerted by muscle bellies are thought to directly correlate to the cross-sectional areas of said muscle bellies [17], several groups have investigated paraspinal muscle atrophy (sarcopenia) as a predictor for PJK [12,13,18,19,20]. In one study, Yagi et al. [20] evaluated the association of psoas and multifidus CSA with the postoperative loss of sagittal alignment and the degree of PJK in 60 degenerative lumbar scoliosis patients treated with instrumented fusion. As in our study, the authors reported a significant correlation between multifidus CSA and changes in the proximal junctional angle (r = 0.22); they also noted a significant correlation between CSA and changes in thoracic kyphosis (r = 0.34). Unlike the present analysis, total paraspinal CSA was not considered, CSA was measured at the L5/S1 disk space [versus UIV], and a multivariable analysis was not performed. Yet, the authors argued that the results support paraspinal musculature atrophy as a risk factor for PJK, and the loss of sagittal plane correction. Pennington and colleagues [6] subsequently described their experience in 169 patients undergoing thoracolumbosacral fusion. Standardizing paraspinal muscle area at the UIV to the vertebral body CSA, they found that relative paraspinal muscularity at the UIV independently predicted PJK risk, along with postoperative positive sagittal balance, and more aggressive sagittal plane correction (measured by ΔPI-LL from pre- to postop). Underlying bone quality in Hounsfield units was not considered in that study, nor was PJK failure mode. Therefore, although they identified UIV sarcopenia as a risk factor for PJK, as in our study, they did not highlight whether it influences failure mode or interacts with underlying bone quality. Additionally, all patients in their study had UIVs at the thoracolumbar junction or lumbar spine. Therefore, it is unclear whether their results would generalize to our population. However, like their study, we did note increased sagittal malalignment trended toward predicting PJK risk; we also noted that poor bone quality was only associated with increased risk of bony (type 2) PJK, so it is possible that they may not have been able to detect the association of bone quality with PJK risk when considering all types of PJK.

Babu et al. [21] subsequently examined psoas muscle CSA as a risk factor for complications following thoracolumbar fusion for ASD incorporating a PSO. Psoas CSA was measured at the L4 level and normalized to the CSA of the L4 body. Using this, the authors reported a significantly lower PJK rate in patients with psoas CSA to vertebral body ratios ≥ 1.1 (0% vs. 34%). So, while they noted an association of decreased muscular CSA with PJK risk, they considered neither postoperative sagittal alignment nor bone quality, as in the present study. Additionally, the psoas plays no biomechanical role in resisting kyphosis about the instantaneous axis of rotation for any motion segment in the mobile spine and it is unclear whether the authors would have seen a similar relationship were paraspinal CSA measured. The present study builds upon this by considering the multifidus and total paraspinal CSA, which contribute to the posterior tension band that resists kyphosis.

Most recently, Pinter and colleagues [12] examined 81 patients from a single center instrumented to the upper thoracic spine (T1-6) for adult spinal deformity. Multivariable logistic regression considering baseline lumbopelvic parameters, underlying bone quality, and fatty infiltration of the multifidus at the mid-L3 body were performed, with patients divided into those with normal muscle quality, moderate fatty infiltration, and severe fatty infiltration based upon Goutalier grade. They reported higher rates of severe fatty infiltration in patients who experienced PJK and reported that lower UIV HU and the presence of moderate or severe fatty infiltration in the multifidus at L3 were independent predictors of PJK by the last follow-up. However, the authors did not account for differences in follow-up time, evaluated the multifidus at the L3 body as opposed to the UIV, and did not evaluate the muscle cross-section area. Similar limitations are noted in an accompanying analysis by the same group [19] that examined PJK following thoracolumbar fusion terminating at the thoracolumbar junction (T10-L2) [19]. Fatty infiltration of the multifidus at L3 and low Hounsfield units at the UIV were both reported to predict elevated risk of PJK. However, CSA as the measure of sarcopenia, CSA of the erector spinae, CSA of the paraspinal musculature at the UIV, and variability in follow-up were not accounted for. As illustrated in the present analysis, total paraspinal CSA appears more predictive than multifidus CSA alone, and CSA at the UIV is more predictive than CSA at L3. The latter also concords with what would be expected biomechanically, given that the paraspinal musculature of the UIV acts on the motion segment at risk of PJK. So, while the present results concord with the findings of the previous authors that suggest poor paraspinal muscle health as a risk factor for PJK, they build upon the prior results by demonstrating that it is specifically the CSA at the UIV that appears to confer risk. Additionally, our results demonstrate low paraspinal CSA as a risk factor specifically for type 1 PJK—discoligamentous or “soft tissue” type PJK. In none of the aforementioned studies was the PJK failure mode examined to evaluate whether the influence of sarcopenia on PJK risk varies by the type of PJK experienced, as was illustrated in the present study.

Given that decreased paraspinal muscle CSA appears to predispose patients to increased odds of PJK, one potential intervention to reduce the risk of discoligamentous PJK is to employ techniques that minimize soft tissue dissection. It is known that open fusion is associated with significant postoperative multifidus muscle atrophy; Hu et al. [22] demonstrated in a rabbit fusion model that open fusion was associated with significant multifidus muscle fiber atrophy in the fusion bed. Weber and colleagues [23] reported similar results in their series of 75 patients who underwent muscle biopsy during either index or revision fusion. Histology demonstrated significant multifidus muscle fiber atrophy and/or alterations in the muscle fiber histology. Radiographically, this can be seen in postoperative MR or CT. As an example, Sihvonen et al. [24] demonstrated decreased paraspinal muscle density in postoperative patients, which corresponded with abnormal EMG results consistent with denervation. Similarly, Cho et al. [25] noted significant decreases in the cross-sectional area of both the multifidus and erector spinae following single-level PLIF. Last, Gejo and colleagues [26] demonstrated that open lumbar surgery was associated with changes in multifidus MR signal, which correlated with retraction time and postoperative decreases in extensor muscle strength. This suggests that postoperative paraspinal muscle atrophy has functional consequences. The use of percutaneous instrumentation is a strategy to decrease postoperative paraspinal muscle atrophy. Kim et al. [27] demonstrated this in their series of 19 patients treated with open (n = 11) or percutaneous instrumentation (n = 8) who underwent pre- and postoperative MR. Patients treated with open fusion had a more than 30% decrease in multifidus CSA, whereas those treated with percutaneous fusion did not have a statistically significant decrease. This also corresponded to a significantly greater increase in extension muscle strength in the percutaneous fusion group. Based on this, it seems reasonable to consider the use of a soft stop at the top of long thoracolumbar fusion constructs terminating in the upper thoracic spine as a mechanism to decrease type 1 PJK risk. This would involve open fusion [as necessary] for the mid-thoracic to the lumbar region and then employing percutaneous fixation at the uppermost 2–3 levels so as to minimize postoperative paraspinal atrophy and thereby reduce type 1 PJK risk.

### 4.2. Bone Density and PJK

Bone quality can be assessed by several metrics [28], though increasingly opportunistic measures such as VBQ and Hounsfield units are gaining attention as they provide the potential to assess the quality of the bone to be instrumented. As with sarcopenia, a handful of studies have evaluated bone quality as a risk factor for PJK. These studies operative on the premise that poor bone quality may result in poor load-sharing capacity and, consequently, failure of anterior column support at the UIV following long-segment fusion. Yagi and colleagues [29] again provided one of the early studies focusing specifically on the association between poor bone quality and the risk of PJK. In the 113 included patients, all of whom underwent ≥5-level fusion for ASD, bone quality was assessed using femoral neck DEXA and dichotomized as mildly low/normal (T-score ≥ −1.5) or significantly low (T-score < −1.5). Using propensity matching of curve type and alignment, the authors noted PJF (ΔPJA ≥ 20°) was 14-fold more common in those with significantly low BMD. While this correlation of low BMD with PJF risk concurs with our results, this earlier study considered neither bone quality of the vertebrae nor paraspinal sarcopenia. PJF type was similarly not considered, though the present results argue that poor quality as a risk factor predominately for type 2 PJK.

Kuo et al. [30] subsequently used VBQ to assess bone quality as a risk factor for PJK in 116 patients undergoing ≥5-level fusion for ASD. On multivariable logistic regression, they found only VBQ to be predictive of PJK, though paraspinal sarcopenia, postoperative alignment, bone quality at the UIV, nor variability in follow-up were considered as in the present study. To this end, a recent study by our own group [31] that did consider follow-up time and UIV bone quality found UIV bone quality to be predictive of PJK, but only when bone quality was measured with HU, not VBQ. Bone quality, as assessed by VBQ, only approached significance, which suggests the need for further validation of the VBQ for predicting mechanical complications following spinal fusion. Contemporaneously Wang and colleagues [32] performed a similar analysis of PJK risk factors in 206 patients who underwent thoracolumbar fusion for adolescent idiopathic scoliosis. As with Kuo and colleagues, Wang et al. [32] found VBQ score to be the only significant predictor of PJK, though again postoperative alignment, follow-up time, nor bone quality [as measured by HU] were considered. Both study results therefore support the present finding associating low HU at the UIV/UIV + 1 with increased PJK risk, but the present study builds upon these by both suggesting that bone quality influences PJK failure mode and suggesting that there is likely an interplay between bone quality and paraspinal muscle integrity for determining PJK failure mode.

In the aforementioned studies by Pinter and colleagues [12,19], HU at the UIV was found to be significantly lower in patients experiencing PJK; however, as mentioned, the authors failed to account for variability in follow-up time and did not consider whether underlying bone quality influences the type of PJK seen. This study attempted to build upon a previous report by Mikula et al. [10], who noted significantly lower mean HU of the UIV/UIV + 1 in patients undergoing long-segment thoracolumbar fusion to the upper thoracic spine (T1-6). They noted an optimal cutoff HU of 159 for the prediction of PJK with an AUC of 0.77, and patients with an average HU < 147 had a nearly 59% higher odds of experiencing PJK or proximal junctional failure (PJF). However, the study was similarly limited by not considering the contribution of the paraspinal musculature or the relative impact of follow-up on PJK odds. The present analysis builds on this by accounting for both factors. It also considers the relative contribution of UIV bone quality and paraspinal sarcopenia to each PJK type. Together, the results suggest that poor bone quality and sarcopenia portend higher PJK risk, though it is likely that sarcopenia may elevate the risk of a more delayed, type 1 (discoligamentous) PJK, whereas poor bone quality predicts a more rapid type 2 (bony) PJK. Furthermore, for the assessment of bony PJK risk, the present results suggest that UIV HU is superior to VBQ.

### 4.3. Limitations

The present study has several limitations. As a retrospective study, it is precluded from determining any causal linkage between paraspinal muscle sarcopenia and either the overall risk of proximal junctional kyphosis following long-segment fusion, or the failure mode/type of PJK observed. Additionally, as a single-center study, the data may reflect institutional biases, such as the decision not to employ soft stops (e.g., polyetheretherketone rods and sublaminar tape) at the rostral end of the construct, which has been suggested to allow for increased motion at the proximal junction and, thus, reductions in stresses on the pedicle screws at the UIV [33,34]. Furthermore, the results note a trend between decreased bone quality of the UIV and UIV + 1 and increased risk of type 2 PJK. However, the overall result was not significant, likely due to the small number of patients who experienced type 2 PJK. This suggests that the present study may be underpowered and would benefit from replication in a larger multicenter cohort. Nevertheless, the association concords with what would be expected from the underlying biomechanical principles though to mediate type 2 PJK, namely the insufficient load-sharing capacity of the UIV or UIV + 1. Another limitation is that the radiographic measures used to determine PJK were performed by unblinded reviewers. This introduces the possibility of observer bias, even though measures were performed by the reviewers independently and the analysis was performed with the averaged values. Last, though muscle contractile power is thought to correlate to CSA, some cadaveric work [17] has suggested that the tendinous insertions of the paraspinal muscles are highly diminutive and so conventional models correlating CSA with contractile power may be inadequate. The present analysis does find total CSA of the paraspinal musculature to predict the risk of type 1 (discoligamentous PJK), which may suggest that radiographic CSA has a criterion but not a construct validity for type 1 PJK. Further investigation is merited. Additionally, future studies should consider the relative impact of the posterior ligamentous complex, including the supraspinous, interspinous, and capsular ligaments, which have been suggested to contribute to biomechanical stability in the trauma literature.

## 5. Conclusions

Proximal junctional kyphosis affects 5–61% of patients surgically treated for adult spinal deformity, with 10–31% of these cases requiring surgical revision. In the present analysis, we found that paraspinal muscle sarcopenia and poor relative bone quality, as assessed by Hounsfield units, may affect both PJK risk and the mode of PJK occurrence. Specifically, paraspinal muscle sarcopenia at the UIV predicts an increased risk of PJK in time-to-event analysis, but in the sub-analysis, this increased risk stems solely from increased risk of discoligamentous (type 1) PJK; the risk of bony PJK (type 2) is not higher in those with sarcopenia. By contrast, poor quality trended toward being more common in those experiencing type 2 PJK, though the present cohort was likely too small to detect the difference as significant. Future investigation using multicenter cohorts is merited.

## Figures and Tables

**Table 1:** Summary of included patients.

	All	PJK	No PJK	*p*
Demographics	76	15	61	
Age (yr)	66.0 [61.0, 71.0]	66 [59, 72]	66 [61, 71]	0.86
Sex (Female)	55 (72.4)	13 (86.7)	42 (68.9)	0.21
Height (m)	1.63 [1.57, 1.72]	1.63 [1.55, 1.65]	1.63 [1.57, 1.73]	0.34
Weight (kg)	74.2 [62.3, 90.3]	68.1 [59.5, 83.9]	77.7 [63.6, 90.7]	0.23
BMI (kg/m^2^)	28.4 [32.6, 24.9]	25.5 [24.9, 30.2]	28.9 [24.9, 32.7]	0.36
mFI5	1 (0, 1.75)	1 (0, 2)	0 (0, 1)	0.09
ASA Class	1 (1, 2)	2 (1, 2)	1 (1, 2)	0.46
CCI	3 (2, 3.75)	2 (2, 4)	3 (2, 3)	0.22
Smoking Status				0.99
Current	5 (6.6)	1 (6.7)	4 (6.6)	
Previous	27 (35.5)	5 (33.3)	22 (36.1)	
None	44 (57.9)	9 (60.0)	36 (47.4)	
Governmental Insurance	44 (57.9)	11 (73.3)	33 (54.1)	0.40
Albumin (g/dL)	4.0 [3.3, 4.4]	4.2 [3.5, 4.4]	3.9 [3.3, 4.3]	0.51
Radiographic follow-up (d)	754 (509, 1296)	594 (217, 1205)	755 (719, 1308)	0.27
Preoperative Radiographics				
SVA (cm)	9.3 [4.1, 13.9]	7.7 [4.3, 12.3]	9.4 [4.0, 14.0]	0.65
PI (°)	54.1 [43.4, 62.9]	57.2 [50.0, 64.6]	53.5 [41.1, 60.9]	0.17
PT (°)	30.1 [23.0, 37.4]	34.1 [25.2, 38.6]	28.9 [22.9, 36.6]	0.22
SS (°)	24.0 [13.2, 31.7]	27.4 [15.6, 34.0]	22.9 [13.0, 31.1]	0.41
L1-S1 LL (°)	31.8 [14.3, 45.6]	31.8 [20.1, 56.5]	31.8 [12.3, 44.2]	0.26
L4-S1 LL (°)	30.8 [23.0, 39.3]	29.5 [26.6, 40.1]	31.2 [23.0, 38.8]	0.78
T1PA (°)	31.6 [23.3, 40.8]	33.5 [25.5, 40.7]	30.0 [22.2, 41.1]	0.76
Global Tilt (°)	61.3 [42.1, 78.6]	57.9 [41.4, 71.3]	62.2 [44.3, 79.9]	0.54
Average HU-UIV	162 [135, 210]	147 [111, 218]	163 [139, 209]	0.31
Average HU-UIV + UIV +1	170 [139, 213]	157 [123, 191]	175 [143, 213]	0.27
VBQ UIV	2.73 [2.35, 3.22]	2.9 [2.7, 3.2]	2.6 [2.4, 3.2]	0.18
Rod Material				0.33
Ti Alloy	20 (26.3)	2 (13.3)	18 (29.5)	
CoCr	56 (73.7)	13 (86.7)	43 (70.5)	
Postoperative Radiographics				
SVA (cm)	2.9 [0.2, 5.8]	4.5 [1.3, 5.9]	2.4 [0.1, 5.6]	0.34
PI (°)	54.3 [46.4, 61.2]	57.3 [49.8, 69.9]	53.4 [46.3, 59.7]	0.16
PT (°)	20.2 [13.8, 26.1]	25.8 [19.2, 29.1]	19.1 [13.4, 24.8]	0.04
SS (°)	35.1 [26.6, 42.0]	35.3 [26.0, 42.1]	33.9 [26.8, 41.6]	0.75
L1-S1 LL (°)	48.5 [40.5, 60.1]	47.1 [35.8, 59.7]	49.3 [41.1, 60.3]	0.56
L4-S1 LL (°)	37.0 [29.7, 43.2]	35.6 [28.4, 41.9]	37.0 [30.0, 43.2]	0.74
T1PA (°)	16.1 [10.1, 23.2]	21.6 [16.1, 28.9]	15.0 [9.5, 21.0]	0.04
Global Tilt (°)	41.1 [24.6, 56.6]	38.6 [27.0, 58.5]	41.5 [22.9, 56.6]	0.49
Morphometrics				
CSA Multifidus-L3 (mm^2^)	485 [295, 607]	346 [295, 490]	543 [311, 632]	0.16
CSA All Paraspinals-L3 (mm^2^)	2869 [2282, 3369]	2589 [1797, 3025]	2993 [2395, 3442]	0.07
CSA Multifidus-UIV (mm^2^)	394 [280, 478]	382 [256, 455]	395 [289, 486]	0.34
CSA All Paraspinals-UIV (mm^2^)	945 [814, 1170]	833 [704, 1050]	964 [859, 1227]	0.04

Key: ASA—American Society of Anesthesiologists; CCI—Charlson Comorbidity Index; cm—centimeter; CSA—cross-sectional area; HU—Hounsfield units; kg—kilogram; m—meter; PI—pelvic incidence; PT—pelvic tilt; SS—sacral slope; SVA—sagittal vertical axis; T1PA—T1-pelvic angle; UIV—upper instrumented vertebrae; VBQ—vertebral bone quality; yr—year.

**Table 2:** Univariable Cox proportional hazards analysis for predictors of PJK with sub-analysis by mode.

	PJK		Type 1		Type 2	
	15		10		4	
Variable	HR [95% CI]	*p*	HR [95% CI]	*p*	HR [95% CI]	*p*
BMI (per kg/m^2^)	0.97 [0.88, 1.08]	0.61	0.94 [0.82, 1.07]	0.32	1.13 [0.92, 1.38]	0.24
UIV						
T1	0.17 [0.01, 3.03]	0.23	0.17 [0.01, 3.02]	0.22	1.01 [0, ∞]	1.00
T2	0.10 [0.01, 1.60]	0.10	0.10 [0.01, 1.76]	0.12	1.01 [0, ∞]	1.00
T3	0.30 [0.03, 2.78]	0.29	0.17 [0.01, 2.00]	0.16	3816 [0, ∞]	0.96
T4	0.18 [0.02, 1.67]	0.13	0.10 [0.01, 1.17]	0.07	2443 [0, ∞]	0.96
T5	0.27 [0.03, 2.51]	0.25	0.23 [0.02, 2.31]	0.21	1.01 [0, ∞]	1.00
T7	Ref	—	Ref	—	Ref	—
Preoperative Radiographics						
Average HU-UIV (per unit)	0.97 [0.99, 1.01]	0.55	1.00 [0.99, 10.2]	0.57	0.99 [0.96, 1.01]	0.22
Average HU-UIV + UIV + 1 (per unit)	1.00 [0.99, 1.01]	0.39	1.00 [0.99, 1.01]	0.72	0.98 [0.96, 1.01]	0.16
Average HU-UIV/UIV + 1 <164	3.05 [1.03, 9.03]	**0.04**	1.55 [0.44, 5.43]	0.49	84.0 [0.03, 2.9 × 10^5^]	0.29
VBQ UIV (per unit)	1.15 [0.85, 1.57]	0.37	1.20 [0.85, 1.70]	0.30	1.10 [0.51, 2.40]	0.81
Postoperative Radiographics						
SVA (per cm)	1.04 [0.92, 1.18]	0.54	1.03 [0.88, 1.20]	0.76	1.02 [0.80, 1.30]	0.87
T1PA (per °)	1.04 [0.99, 1.10]	0.15	1.01 [0.95, 1.08]	0.72	1.12 [0.99, 1.26]	0.07
Global Tilt (per °)	1.03 [1.00, 1.05]	0.09	1.03 [0.99, 1.06]	0.16	1.00 [0.96, 1.06]	0.88
Morphometrics						
CSA All Paraspinals-L3 (per 100 mm^2^)	0.94 [0.89, 1.01]	0.07	0.94 [0.87, 1.02]	0.12	0.99 [0.89, 1.09]	0.79
CSA All Paraspinals-UIV (per 100 mm^2^)	0.74 [0.57, 0.96]	**0.02**	0.64 [0.45, 0.92]	**0.02**	0.82 [0.55, 1.21]	0.32
Albumin (per g/dL)	1.13 [0.47, 2.73]	0.79	1.01 [0.36, 2.84]	0.99	1.31 [0.20, 8.44]	0.78

Key: cm—centimeter; CSA—cross-sectional area; HU—Hounsfield units; SVA—sagittal vertical axis; T1PA—T1-pelvic angle; UIV—upper instrumented vertebrae; VBQ—vertebral bone quality. **Bolded** values in the table indicated statistical significance at the *p* < 0.05 level.

**Table 3:** Multivariable Cox proportional hazards model for predictors of any PJK.

Variable	HR	95% CI	*p*
Postop Global Tilt	—	—	0.22
Average HU-UIV + UIV + 1 (per unit)	2.98	[0.99, 8.92]	0.05
CSA All Paraspinals-L3 (per 100 cm^2^)	—	—	0.10
CSA All Paraspinals-UIV (per 100 cm^2^)	0.74	[0.57, 0.96]	0.03

Key: cm—centimeter; CSA—cross-sectional area; UIV—upper instrumented vertebra.

**Table 4:** Comparison of patients who experienced type 1 and type 2 proximal junctional kyphosis following long-segment thoracolumbar fusion for adult spinal deformity with an upper instrumented vertebra in the upper thoracic spine (T1-5).

Variable	Type 1	Type 2	*p*
Time to PJK (d)	932 [419, 1452]	93 [129, 287]	0.14
Average HU UIV	168 [106, 270]	140 [130, 150]	0.54
Average HU UIV + 1	174 [142, 249]	130 [141, 152]	0.11
Average HU UIV and UIV +1	177 [124, 266]	140 [130, 151]	0.30
UIV VBQ	3.0 [2.5, 3.7]	2.8 [2.5, 3.5]	0.81
Postoperative SVA (cm)	4.66 [−1.06, 5.89]	3.88 [2.38, 4.97]	0.84
Postoperative T1PA (°)	18.5 [14.0, 23.2]	26.2 [21.8, 29.1]	0.24
Global tilt (°)	38.0 [27.0, 58.5]	43.7 [29.1, 55.7]	0.99
CSA All Paraspinals-L3 (cm^2^)	340 [295, 490]	404 [295, 679]	0.84
CSA Multifidus-L3 (cm^2^)	2637 [1797, 3019]	2834 [2371, 3321]	0.54
CSA All Paraspinals-UIV (cm^2^)	327 [239, 423]	419 [319, 461]	0.30
CSA Multifidus-UIV (cm^2^)	795 [704, 922]	866 [714, 1011]	0.84

Key: CSA—cross-sectional area; d—day; HU—Hounsfield unit; T1PA—T1-pelvic angle; UIV—upper instrumented vertebra; VBQ—vertebral bone quality.

## Data Availability

The original contributions presented in the study are included in the article, further inquiries can be directed to the corresponding authors.

## Abbreviations

cm	centimeter
CSA	cross-sectional area
HU	Hounsfield units
mm	millimeter
PI	pelvic incidence
PT	pelvic tilt
SS	sacral slope
SVA	sagittal vertical axis
T1PA	T1-pelvic angle
UIV	upper instrumented vertebrae
VBQ	vertebral bone quality
yr	year
